# Role of atrial wall thickness in wave-dynamics of atrial fibrillation

**DOI:** 10.1371/journal.pone.0182174

**Published:** 2017-08-21

**Authors:** Jun-Seop Song, Jin Wi, Hye-Jeong Lee, Minki Hwang, Byounghyun Lim, Tae-Hoon Kim, Jae‐Sun Uhm, Boyoung Joung, Moon‐Hyoung Lee, Jeong-Wook Seo, Hui-Nam Pak

**Affiliations:** 1 Yonsei University Health System, Seoul, Republic of Korea; 2 Department of Pathology, Seoul National University College of Medicine, Seoul, Republic of Korea; University of Tampere, FINLAND

## Abstract

**Background/Aims:**

Atrial anatomy and thickness may affect the electrical wave-dynamics of atrial fibrillation (AF). We explored the relationship between left atrial (LA) wall thickness (LAWT) or LA geometry and AF wave-dynamics.

**Methods:**

We included 15 patients with persistent AF (age, 62.3 ± 11.9 years) who underwent AF catheter ablation. We measured the LAWT, LA endocardial curvature, and SD-curvature (surface bumpiness) from preprocedural computed tomography images. We compared those anatomical characteristics with electrophysiologic parameters such as dominant frequency (DF), Shannon entropy (ShEn), or complex fractionated atrial electrogram (CFAE)-cycle length (CL), calculated from intracardiac bipolar electrograms (300–500 points, 5 s), acquired during ablation procedures.

**Results:**

1. LAWT (excluding fat) varied widely among patients, locations, and types of AF. LAWT was inversely correlated with LA volume (r = -0.565, p = 0.028) and positively correlated with SD-curvature (r = 0.272, p < 0.001). 2. LAWT was positively correlated with ShEn (r = 0.233, p < 0.001) and negatively correlated with CFAE-CL (r = -0.107, p = 0.038). 3. In the multivariate linear regression analyses for AF wave-dynamics parameters, DF (β = -0.29 [95% CI -0.44–-0.14], p < 0.001), ShEn (β = 0.19 [95% CI 0.12–0.25], p < 0.001), and CFAE-CL (β = 7.49 [95% CI 0.65–14.34], p = 0.032) were independently associated with LAWT.

**Conclusion:**

Regional LAWT is associated with LA structural features, and has significant correlations with the wave-dynamics parameters associated with electrical wavebreaks or rotors in patients with persistent AF.

## Introduction

Atrial fibrillation (AF) is a progressive disease. Recurrent or sustained AF causes electrical and structural remodeling of the atrium, making it difficult to restore and maintain sinus rhythm [1]. AF progression and related atrial remodeling can result in the dilatation of the left atrium (LA), fibrosis, and hypertrophy [2, 3]. Such structural changes in atrial tissue have been known to affect electrical conduction, electrophysiologic characteristics, and electrical wave-dynamics during fibrillation [4, 5]. Animal studies suggested that the presence of interstitial fibrosis is associated with slow conduction and conduction block within the atria, and consequently facilitates the induction of AF through local reentry. Moreover, although the atrial wall is thin, it has a multilayer regional difference of conduction and repolarization. Such transmural endo-epicardial asynchrony plays some role in continuous reentry and AF maintenance [6]. Konings et al. also described wave collision, conduction block, pivot point of rotational activity, and slow conduction in the atria as potential mechanisms for complex fractionated atrial electrograms (CFAEs) [7]. Therefore, it is clear that atrial wall thickness, atrial morphology, or degree of histological changes contribute to the electrophysiological characteristics and wave-dynamics of AF. Although the role of endo-epicardial dissociation and atrial bundle rearrangement in the progression of human AF has been emerged [8, 9], only a few systemic exploration studies demonstrate the relationship between such geometrical changes of LA and electrophysiological results in AF. Therefore, we hypothesized that left atrial wall thickness (LAWT) and LA wall geometry are associated with the AF wave-dynamics. We evaluated LAWT and LA endocardial surface bumpiness (SD of curvature) from cardiac computed tomography (CT) images of the patients. We also investigated multiple parameters reflecting AF wave-dynamics reflecting multiple wavelets [10] or focal sources [11] from clinically acquired bipolar electrograms during the AF ablation procedure.

## Materials and methods

### Study population

The study protocol adhered to the Declaration of Helsinki and was approved by the Institutional Review Board of the Yonsei University Health System. All subjects provided written informed consent for the use of their cardiac CT images and intracardiac electrograms. The present study included a total of 15 patients with nonvalvular persistent AF (PeAF) who underwent radiofrequency catheter ablation (RFCA) for drug-refractory AF. All patients maintained optimal anticoagulation levels (target INR 2.0–3.0) before the procedure, and antiarrhythmic drugs were discontinued for at least five half-lives of each drug. None of the patients used amiodarone.

### Cardiac CT

We performed contrast-enhanced cardiac CT (Somatom Definition Flash; Siemens Healthcare, Forchheim, Germany) within 2 days before RFCA. We injected the contrast (Iopamiro 370; Bracco, Milan, Italy) into the antecubital vein (flow rate of 5 mL/s) by using a triple-phase method (60–80 mL pure contrast, 30 mL 7:3 saline-to-contrast mixture, and 20 mL pure saline). The test-bolus technique was used to determine the scan delay time. Scanning was performed to target end-systolic phase with prospective electrocardiogram-gated axial acquisition by using the absolute delay method [12]. Cardiac CT images were reconstructed with a slice thickness of 0.75 mm and an interval of 0.5 mm.

### LA volume measurement by using cardiac CT

We estimated LA volume by automatically tracing the LA borders on three-dimensional (3D) LA reconstruction from CT images [13]. We detected the endocardial border based on a Hounsfield unit with additional manual correction. The LA appendage (LAA) was included; however, pulmonary veins (PVs) at their ostia and the mitral annulus at the insertion point of the mitral valve leaflets were excluded from the analysis.

### LAWT measurement with cardiac CT

**Fig 1:**
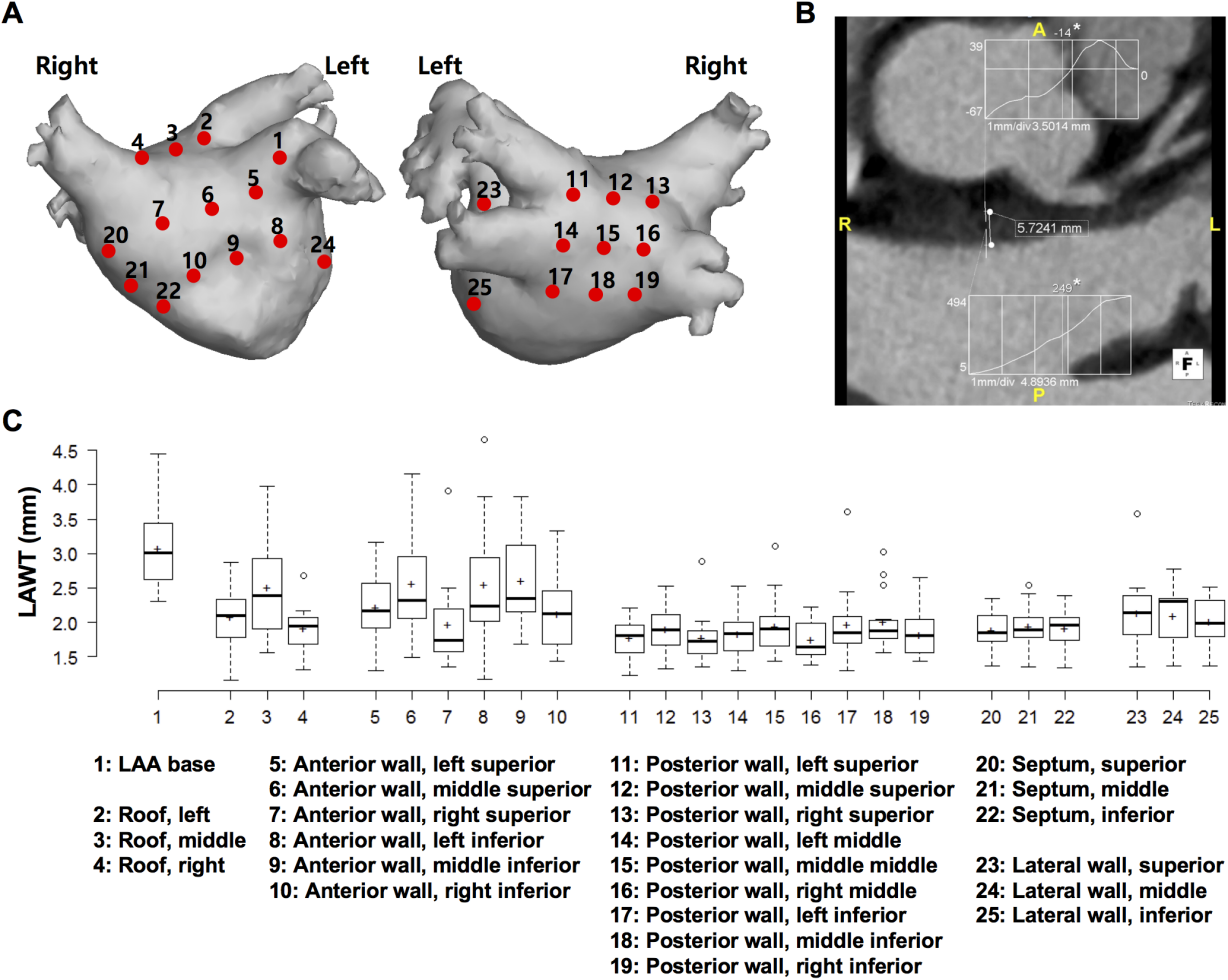
Measurement of LAWT at 25 preselected left atrial locations. (A) Schematic representation of the 25 preselected left atrial locations including 3 at the atrial roof, 1 at the anterior appendage base, 6 at the anterior wall, 9 at the posterior wall, 3 at the septum, and 3 at the lateral wall. (B) Measurement of LAWT at the middle superior anterior wall. LAWT was measured semi-automatically between the inner and outer borders through a histogram and a line segment tool by using software. In inset graphs, the numbers in the y-axes denote the extreme computed tomography (CT) numbers of the two short lines across the LA wall and epicardial fat and LA wall and LA cavity. Numbers marked with an asterisk represent the median CT numbers from the full width at half-maximum method, which correspond with the dots on the line across the LA wall. (C) Standard box plots of regional LAWT with average LAWT (marked by “+”) are shown for each preselected location. LA, left atrium; LAA, left atrial appendage; LAWT, left atrial wall thickness.

All parameters including LAWT were obtained at 25 preselected locations in six regions, including the LAA base, roof, anterior wall, posterior wall, septum, and lateral wall, and agreed on between the radiologists and cardiologists (Fig 1A) [14]. The roof was defined as the most cranial part of the LA, connecting the upper aspect of the venoatrial junctions of the right and left superior PVs. The LAA base was defined as the anterior portion of the LAA neck, within 5 mm of the LA-LAA junction. The septum referred to the interatrial muscular wall that separates adjacent atrial chambers. We divided the regions into superior/middle/inferior and left/middle/right portions on the LA surface, and determined a reference point as a center of each portion in every patient. We averaged the LAWTs measured at 10 points within 5 mm of each reference point.

CT images were analyzed with Aquaris Intuition 4.4.6 software (Terarecon, San Francisco, CA, USA). Two radiologists (with 8 and 11 years of experience in cardiac CT), blinded to the clinical and electrophysiological data, independently evaluated the CT images. We measured the LAWT on the multiplanar reformatted axial, sagittal, and coronal images to obtain perpendicular length, and applied a semi-automated algorithm developed by Wi et al. [15] (Fig 1B). At the preselected point, two short vertical lines were drawn and the border points of the LA wall were automatically estimated across the LA wall and epicardial fat, and across the LA wall and LA cavity by using the CT attenuation difference. LAWT was automatically measured between the inner and outer margins.

### Calculation of the curvature and bumpiness of LA geometry

We reconstructed the LA endocardial geometry from the 3D spiral CT by using the NavX system. The triangular mesh was HC-Laplacian smoothed to remove noise, using MeshLab 1.3.3 software. The LA mesh was uniformly resampled with ACVD software.

**Fig 2:**
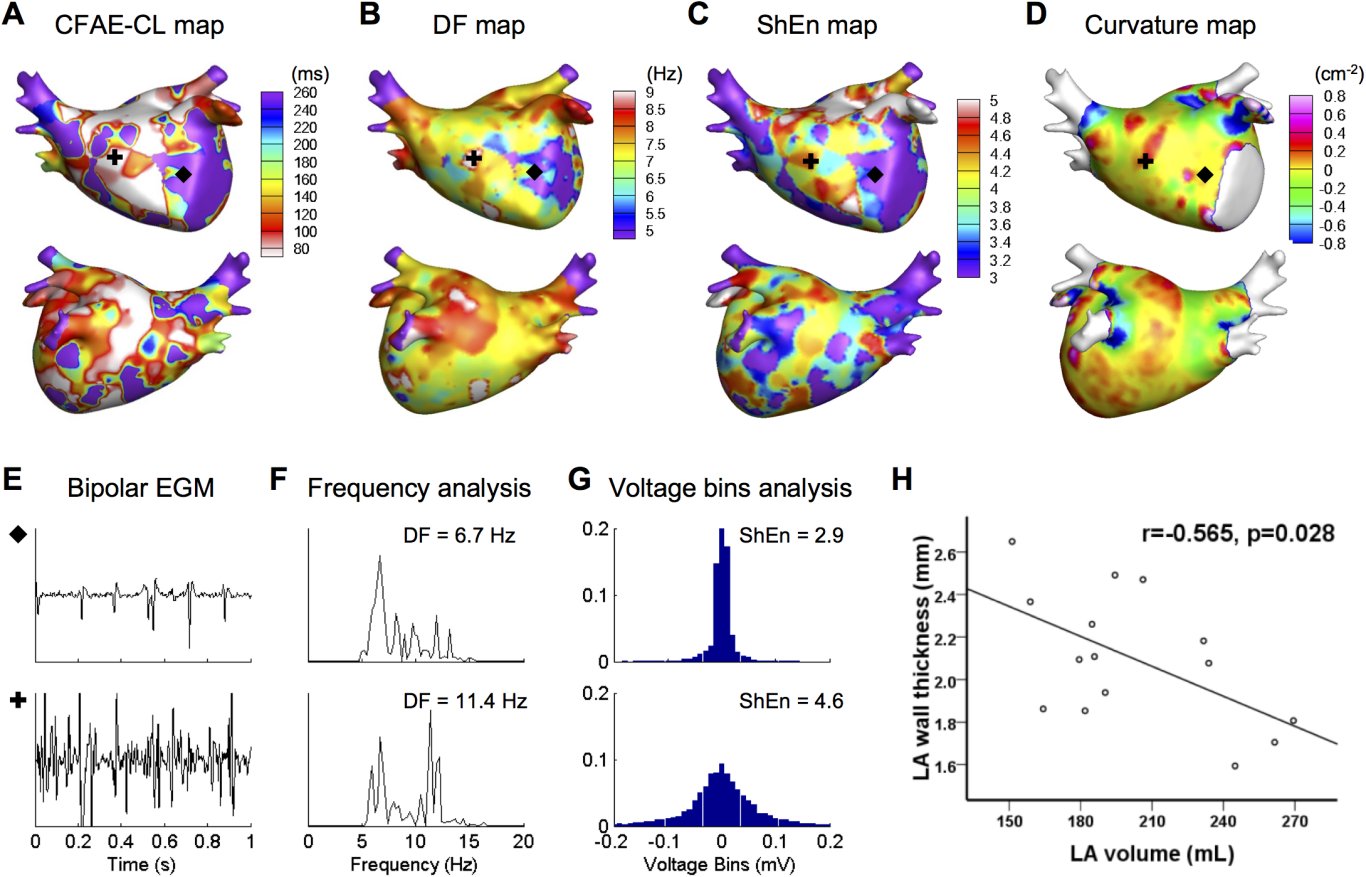
Three-dimensional color-coded mapping of AF wave-dynamics parameters and LA geometric parameter. (A) CFAE-CL, (B) DF, (C) ShEn, and (D) curvature maps. (E–G) Examples of fractionated and unfractionated EGM and their analyses. The frequency spectrum (F) and voltage histogram (G) were analyzed to compute the DF and ShEn of bipolar EGMs, respectively (recording sites are marked by black symbols). (H) An inverse relationship between mean LA wall thickness and LA volume. CFAE-CL, complex fractionated atrial electrogram-cycle length; DF, dominant frequency; ShEn, Shannon entropy; EGM, electrogram; LA, left atrium.

At each point of the LA mesh, we calculated the Gaussian curvature (briefly, “curvature”) by applying the angle deficit method (Fig 2D) [16]. To quantify the degree of bumpiness of the LA surface, we defined the surface bumpiness as the standard deviation (SD) of the curvature as follows:

$$\sqrt{\frac{1}{S}\iint {\left(K-\bar{K}\right)}^{2}\;dA}$$

where *S* is the surface area, *K* is the curvature, and $\bar{K}$ is the average of the curvature on the surface. The SD-curvature (bumpiness) represents the heterogeneity of the curvature (i.e., how bumpy the tissue is). In the 25 regions of the LA, we calculated the average of the curvature and the SD-curvature. The C++ code was implemented for the analysis.

### LAWT measurement in cadaveric hearts

A postmortem analysis of LAWT was performed in 17 human heart specimens (cadaveric hearts), none of which had a history of AF, using calipers. We measured the LAWT at 24 preselected locations, including the roof, anterior wall, posterior wall, septum, and lateral wall. The LAA base was excluded owing to technical limitations.

### Electrophysiological mapping and assessment of AF wave-dynamics

Intracardiac bipolar electrograms were recorded by using the Prucka CardioLab Electrophysiology system (General Electric Health Care System Inc., Milwaukee, WI, USA). Electroanatomical mapping was performed with the NavX system (St. Jude Medical Inc., Minneapolis, MN, USA). By using a multipolar ring catheter (Lasso; Johnson & Johnson Inc., Diamond Bar, CA, USA), 5-s bipolar electrograms were recorded at well-distributed throughout the LA during AF. The signals were exported at a sampling rate of 2.0 kHz and band-pass filtered from 32 to 300 Hz.

We determined the AF wave-dynamics parameters, including the CFAE-cycle length (CL), dominant frequency (DF), and Shannon entropy (ShEn) of the bipolar electrograms acquired from >350 points on the entire LA (Fig 2E–2G). To generate a CFAE-CL map, we calculated CFAE-CL as the average time duration between consecutive deflections, which were identified by the downstroke morphology between the local-maximum and the local-minimum amplitudes. CFAEs were defined as those with CFAE-CL <120 ms [17]. We used a peak-to-peak criterion of 0.03 mV to exclude baseline noise. Additionally, we set a refractory period of 40 ms to avoid multiple detections of a single deflection event and an electrogram width of 15 ms to exclude the detection of the far-field event [18].

We performed spectral analysis of the bipolar electrograms to determine the DF [19]. The bipolar signals were tapered at their edges to a 0 value by using the Hanning window, rectified, and band-pass filtered from 3 to 15 Hz. We performed an 8192-point fast Fourier transformation with a spectral resolution of 0.24 Hz. The DF was defined as the frequency with the maximum amplitude at that site for each signal [20]. To ensure reliability in DF detection, we calculated the regularity index (RI), defined as the ratio of the power at the DF and its adjacent frequencies (= 0.75-Hz band) to the power of the 3- to 15-Hz band [21]. Points with RI of <0.2 were excluded.

The ShEn of the bipolar electrogram was calculated by using the method of Ganesan et al. [22] The voltage histogram of the bipolar signal was generated with 0.01-mV fixed amplitude bins. At each bin, the relative probability density was calculated by dividing the number of counts in that bin by the total number of counts in all bins. Then, the ShEn was defined as follows:

$$-\sum_{i=0}^{N-1}P_{i}\log_{2}P_{i}$$

where *N* is the total number of bins and *P*_*i*_ is the relative probability density of each bin.

We developed a MATLAB-based custom software for the signal analysis (MathWorks, Natick, MA, USA).

### Statistical analysis

Continuous variables are expressed as mean ± SD, and categorical data are shown as absolute values and percentages. Continuous variables were compared by using the Student’s t-test or ANOVA. Categorical variables were compared with the Chi-square test or Fisher’s exact test. The degree of linear correlation was analyzed by using Pearson correlation coefficient. To study whether there was an independent relationship between LAWT and AF wave-dynamics parameters, we performed multiple regression analysis by using forward stepwise selection. We calculated the intraclass correlation coefficient (ICC) to evaluate the consistency between two observers in LAWT measurements. A p-value of less than 0.05 was considered as statistically significant. Statistical analysis was performed using SPSS 22.0 (SPSS Inc., Chicago, IL, USA).

## Results

### Baseline characteristics

Table 1 shows the baseline clinical characteristics. A total 15 patients with PeAF (11 men; mean age, 62.3 ± 11.9 years) were included in this study. The mean CHA_2_DS_2_-VASc score was 2.5 ± 2.1. The mean left ventricular ejection fraction was 60.8 ± 10.2%, and the ratio of early diastolic transmitral flow velocity to peak diastolic tissue velocity was 12.0 ± 6.2 on echocardiography. The mean LA volume was 202.6 ± 37.2 mL.

**Table 1:** Baseline characteristics of patients.

Variables	Persistent AF (n = 15)
Male	11 (73%)
Age, years	62.3 ± 11.9
65–74	3 (20%)
≥75	2 (13%)
Hypertension	7 (47%)
Diabetes	5 (33%)
Heart failure	0 (0%)
Stroke	3 (20%)
Vascular disease	4 (27%)
CHA_2_DS_2_-VASc score	2.5 ± 2.1
LA volume, mL	202.6 ± 37.2
LVEF, %	60.8 ± 10.2
E/Em	12.0 ± 6.2

AF, atrial fibrillation; LA, left atrium; LVEF, left ventricular ejection fraction; E/Em, ratio of peak velocity of early diastolic mitral inflow and early diastolic mitral annular velocities.

### Regional variability of LAWT and association with LA geometry

The measurements of LAWT by using CT attenuation differences showed excellent agreement between two independent observers (ICC = 0.979, p < 0.001). Significant differences in LAWT were observed among the 25 preselected locations (Fig 1C). There was a large interpatient variation in mean LAWT (range, 1.59–2.65 mm), and overall mean LAWT was 2.10 ± 0.60 mm. As shown in Table 2, there was a significant regional variability in LAWT (p < 0.001). The LAA base had the thickest wall and the posterior wall was the thinnest (3.13 ± 0.74 vs. 1.86 ± 0.40 mm, p < 0.001). The anterior wall (2.34 ± 0.72 mm) and roof (2.17 ± 0.59 mm) were thicker than the mean LAWT, whereas the septum (1.92 ± 0.30 mm) and lateral wall (2.08 ± 0.43 mm) were thinner. In addition, structurally normal cadaveric heart specimens showed a similar tendency of LAWT distribution to AF (Table 2). While the anterior wall was the thickest, the posterior wall was the thinnest in cadaveric specimens (2.13 ± 0.61 vs. 1.15 ± 0.84 mm, p < 0.001).

**Table 2:** Comparisons of parameters according to the LA regions.

	LAWT	LAWT(Cadaver)	DF	ShEn	CFAE-CL	CFAE	Curvature	Bumpiness
	(mm)	(mm)	(Hz)		(ms)	(n, %)	(cm^-2^)	(cm^-2^)
Total	2.10±0.60	1.52±0.82	6.76±0.95	4.00±0.64	151.75±60.54	136 (36%)	-0.05±0.20	0.31±0.38
LAA base	3.13±0.74	N/A	6.92±1.24	4.63±0.72	129.95±45.33	8 (53%)	-0.37±0.47	1.72±0.33
Roof	2.17±0.59	1.89±0.90	6.79±0.95	3.98±0.66	159.45±67.14	12 (27%)	-0.18±0.19	0.36±0.23
Anterior wall	2.34±0.72	2.13±0.61	6.61±0.93	3.93±0.65	153.87±62.37	30 (33%)	-0.02±0.12	0.23±0.19
Posterior wall	1.86±0.40	1.15±0.84	6.92±0.99	3.99±0.57	145.44±53.77	57 (42%)	-0.06±0.16	0.20±0.22
Septum	1.92±0.30	1.59±0.67	6.57±0.81	3.86±0.65	150.85±60.71	15 (33%)	0.04±0.08	0.20±0.13
Lateral wall	2.08±0.43	1.92±0.75	6.73±0.89	4.14±0.63	166.88±70.98	14 (31%)	0.05±0.18	0.36±0.37
p	<0.001	<0.001	0.147	0.001	0.215	0.238	<0.001	<0.001

LA, left atrium; LAA, left atrial appendage; LAWT, left atrial wall thickness; CFAE, complex fractionated atrial electrograms; CL, cycle length; DF, dominant frequency; ShEn, Shannon entropy.

Additionally, LAWT was associated with other LA geometric features. We observed that the mean LAWT over the entire LA was inversely associated with the LA volume (r = -0.565, p = 0.028; Fig 2H). Furthermore, LAWT was positively correlated with SD-curvature (bumpiness) of the 25 preselected LA regions (r = 0.272, p < 0.001).

### Relationship between LAWT and AF wave-dynamics parameters

We determined AF wave-dynamics of 15 patients with PeAF by calculating the wave-dynamics parameters from intracardiac bipolar electrograms acquired during AF (Fig 2A–2C). Table 2 shows the regional variability of AF wave-dynamics parameters as well as the LA structural parameters. The LAA base, which is the thickest region of the LA, showed the shortest CFAE-CL and the highest DF, ShEn, and CFAE over the entire LA.

LAWT was significantly associated with all wave-dynamics parameters. The linear correlation analyses showed that LAWT was positively correlated with ShEn (r = 0.233, p < 0.001) and negatively correlated with CFAE-CL (r = -0.107, p = 0.038). Additionally, there were pairwise linear relationships among the parameters of AF wave-dynamics. DF showed a positive correlation with ShEn (r = 0.454, p < 0.001) and a negative correlation with CFAE-CL (r = -0.380, p < 0.001), and ShEn showed a negative correlation with CFAE-CL (r = -0.760, p < 0.001).

To determine whether LAWT and AF wave-dynamics parameters influence the other wave-dynamics parameters, we performed stepwise multiple linear regression analyses (Table 3). All wave-dynamics parameters, including DF (β = -0.29 [95% CI -0.44–-0.14], p < 0.001), ShEn (β = 0.19 [95% CI 0.12–0.25], p < 0.001), and CFAE-CL (β = 7.49 [95% CI 0.65–14.34], p = 0.032), were independently associated with LAWT.

**Table 3:** Stepwise linear regression analyses for the parameters representing AF wave-dynamics.

	Univariate			Multivariate		
	β	95% CI	p	β	95% CI	p
**DF**						
LAWT	-0.10	-0.27–0.06	0.206	-0.29	-0.44–-0.14	<0.001
ShEn	0.68	0.54–0.82	<0.001	0.74	0.61–0.88	<0.001
CFAE-CL	-0.01	-0.01–-0.00	<0.001			
**ShEn**						
LAWT	0.25	0.14–0.35	<0.001	0.19	0.12–0.25	<0.001
DF	0.30	0.24–0.36	<0.001	0.14	0.10–0.19	<0.001
CFAE-CL	-0.01	-0.01–-0.01	<0.001	-0.01	-0.01–-0.01	<0.001
**CFAE-CL**						
LAWT	-10.86	-21.11–-0.62	0.038	7.49	0.65–14.34	0.032
ShEn	-72.36	-78.65–-66.07	<0.001	-74.00	-80.44–-67.56	<0.001
DF	-24.11	-30.09–-18.13	<0.001			

DF, dominant frequency; LAWT, left atrial wall thickness; ShEn, Shannon entropy; CFAE-CL, complex fractionated atrial electrogram-cycle length.

## Discussion

### Main findings

In the present study, we investigated the role of LAWT and LA geometry in human AF wave-dynamics across points over the entire LA by utilizing sophisticated computational methods. The first major finding is that the range of LAWT varied, and significant interpatient and intra-patient regional variabilities in LA geometry and AF wave-dynamics parameters, as well as in LAWT, were observed. Second, the LAWT was inversely associated with the LA volume and positively correlated with regional SD-curvature (bumpiness). Third, LAWT was correlated with wave-dynamics parameters including DF, ShEn, or CFAE-CL, and the major independent determinant of AF wave-dynamics parameters in the multiple regression model. In this study, we demonstrate the relationship between LAWT and electrophysiological map of wave-dynamics parameters.

### LAWT and LA geometry in patients with AF

The LA wall is a thin structure; however, it has nonuniform thickness with considerable regional heterogeneity. We measured the LAWT by using customized software in this study, and validated the regional LAWT pattern in human cadaveric heart specimens despite the lack of AF history and the limitation in using formalin-preserved tissue. The LAA base and anterior wall were thicker than other areas, whereas the posterior wall was the thinnest, consistent with a prior study involving conventional LAWT measurements in PeAF [23]. It has been known that a long-lasting AF results in progressive remodeling of the LA and different aspects of regional variability in LAWT, which were observed in the current study and other studies [24, 25]. Moreover, LAWT was inversely associated with LA volume, which implies that LA structural remodeling is excessively advanced in thin-walled areas and has relatively little contact with rigid extracardiac structures according to Laplace’s law. However, the LA remodeling process is not a simple geometrical change, and multiple histopathological processes, hemodynamic factors, genetic factors, or electrophysiological factors may contribute to a higher incidence of non-PV foci and AF maintenance mechanisms [26, 27].

### Role of LAWT in AF maintenance mechanisms

The intramural conduction between endocardial and epicardial layers facilitates pro-arrhythmogenic transmural dynamics, such as abrupt breakthrough and local intramural reentry [6, 28]. This endo-epicardial electrical dissociation is closely related to LAWT [9] and structural remodeling of atrial wall [29]. Additionally, the heterogeneous wall thickness induces spiral wave localization or drift [30, 31], and the curvature change of tissue geometry promotes initiation and maintenance of reentries by promoting wavebreaks [32]. In this study, we calculated DF, ShEn, and CFAE of clinically-acquired bipolar electrograms, which reflect the focal source [19, 22] and/or wavebreak [33] mechanisms of AF. We also demonstrated their relationship with LAWT and LA geometry, despite the existence of ongoing debates regarding uncertain complex mechanisms of AF and their influences on bipolar electrograms [34, 35]. The present study also revealed close relationships among AF wave-dynamics parameters such as DF, ShEn, and CFAE.

### Clinical implications

The importance of imaging modalities in the treatment of cardiac arrhythmias is rapidly growing. Cardiac imaging is starting to play a crucial role in demonstrating cardiac remodeling with tissue characteristics, beyond just showing cardiac structures. Therefore, it provides valuable information in the invasive treatment of complex arrhythmias such as AF or ventricular tachycardia [36]. Additionally, translational applications of image-based computer simulation are increasing [37, 38]. In this study, we delineated the margin of the LA wall and measured the LAWT accurately and reproducibly by applying our semi-automated quantitative method for analyzing the CT attenuation difference, in contrast to conventional direct measurement through manual assessment [39]. We also demonstrated that the LAWT and LA geometry evaluated using CT images were closely associated with multiple wave-dynamics parameters, such as DF, ShEn, or CFAE-CL, generated from clinically acquired intracardiac electrograms. Therefore, our computational methods for detecting regional wall thickness, geometry, and electrophysiologic parameters will provide potential information in determining the wave-dynamics of human AF.

### Limitations

This study has several limitations. First, this study had a small sample size of patients with PeAF. Second, LA size is dynamic as it changes with contraction and relaxation, which might affect the LAWT. Third, the shapes and geometries of the LA may be different among patients owing to variabilities in the atrial remodeling process. To resolve this problem, the measurement points were set relative to the shape of the LA in each patient. Fourth, measuring LAWT using CT images has fundamental uncertainty due to poor soft-tissue contrast of CT scan. Also, we did not display LAWT map in this study, because of low spatial resolution of LAWT data (25 points) compared to other AF wave-dynamics maps (300~500 points). However, LAWT measured using CT images showed similar tendency with that found in the cadaveric heart specimens, and other current techniques for measuring LAWT still showed large variability each other [39]. The recently developed imaging technology for high-resolution mapping of LAWT on the entire LA [40] might provide more detailed information about the spatial heterogeneity of LAWT. Finally, the bipolar electrograms did not exactly represent complex electrical wave dynamics on the entire LA, since they were sequentially obtained over the entire LA, and affected by catheter [41]. Further direct high-density mapping studies would be helpful to clearly reveal the currently uncertain AF mechanisms such as ectopic source, rotor, multiple wavelet, and endo-epicardial dissociation [35].

## Conclusion

LAWT has significant correlations with LA geometry and plays significant roles in human AF wave-dynamics, including electrical wavebreaks and rotors. In addition, there are significant regional differences in LAWT, LA geometric parameters, and AF wave-dynamics parameters.
